# HLA‐B*44:138Q: Evidence for a confined deletion and recombination events in an otherwise unaffected HLA‐haplotype

**DOI:** 10.1111/tan.13439

**Published:** 2019-01-06

**Authors:** Ingrid Faé, Sabine Wenda, Cornelia Grill, Gottfried F. Fischer

**Affiliations:** ^1^ Department for Blood Group Serology and Transfusion Medicine Medical University of Vienna Vienna Austria; ^2^ Department for Blood Group Serology and Transfusion Medicine General Hospital of Vienna Vienna Austria

## Abstract

We discovered a new HLA‐B allele, HLA‐B*44:138Q, and confirmed its segregation. For characterisation, we used serology, sequence specific oligonucleotide (SSO), sequence specific primer (SSP), and full length sequencing by Sanger and next‐generation sequencing. From an evolutionary point the 5′ part of the new allele is identical with alleles from the HLA‐B*44:02 group, while its 3′ part is identical to the HLA‐B*15:18:01:02 allele, the breakpoint being located somewhere between intron 3 and exon 4. The salient feature of the new allele is a deletion of codon 94 in exon 3, which is unique for HLA‐alleles reported so far. Gene conversion can be hypothesised in the generation of this HLA sequence; however, the deletion seems to have occurred additionally. Other HLA‐alleles of the new allele's haplotype were common alleles.

1

Low resolution HLA‐typing of a patient indicated the existence of a novel allele. We proceeded by performing high‐resolution typing by sequencing. Because the characterisation of the new allele required full length sequence analysis, we performed Sanger sequencing on cloned long‐range polymerase chain reaction (PCR) products or amplicons generated by allele specific primers. The advent of next‐generation sequencing (NGS) allowed us to assess the comparability of both methods in respect to effort and reliability of data on this new sequence. Biochemical tests for expression of the new allele were impossible because of lack of material. However, serological analyses were applied to check the expression of HLA epitopes on the cell surface.

Both techniques, SSO and SSP, led to inconclusive results in the HLA‐B typing of an human immunodeficiency virus‐positive patient; the most probable genotype seemed HLA‐B*07,*44. Subsequent Sanger sequencing of exons 1, 2, and 3 revealed a heterozygous genotype consisting of an HLA‐B*07:02, and a novel HLA‐B*44 related allele; thus, refining the initial findings. The sequence of the new allele could be perfectly aligned with HLA‐B*44:02:01:01; there was a deletion of three bases at codon 94, although.

To exclude phasing artefacts, we extended the analyses by cloning and sequenced 20 clones spanning exons 1 to 3. By this strategy, the results from the heterozygous sequencing were confirmed.

To characterise the novel allele more comprehensively, we amplified the whole novel allele by long‐range PCR using allele specific primers located in the 5′ and 3′ end of the gene. For Sanger sequence analyses, we used 11 sequencing primers (all oligonucleotides of this study are listed in Table 1) that created overlapping sequences of forward and reverse strands.

**Table 1:** Oligonucleotides used for sequencing based HLA typing with Sanger or next‐generation sequencing (NGS) technology

Designation	Sequence (5′‐ >3′)	Coverage	Reference	Sequencing technology
A. Primers used for polymerase chain reaction (PCR)‐amplification				
5BIN1CG*	CGG GGG CGC AGG ACC CGG	HLA‐B, Int1 ‐ Ex3	1	Sanger
3BIn3‐37*	AGG CCA TCC CCG SCG ACC TAT		1	
CL1‐14AMp‐B1*	CGA GGA TGC GGG TCA CGG C	HLA‐B, Ex1‐Ex2	In house	Sanger
CL1‐320G rev*	CCT CGC TCT GGT TGT AGT AGC		In house	
B*44‐‐18fwd*	GCA CCC ACC CGG ACT CAG AA	HLA‐B, full length	In house	Sanger
B*44‐4347rev*	GGG GTC ACG GTG GAC ACG G		In house	
A‐F1fwd	AAC TCA GAG CTA AGG AAT GAT GGC AAA T	HLA‐A, full length	2	NGS
A‐F2 fwd	AAC TCA GAG CTA TGG AAT GAT GGT AAA T		2	
A‐R1 rev	ATA TAA CCA TCA TCG TGT CCC AAG GTT C		2	
B‐5’UTR fwd	GGC AGA CAG TGT GAC AAA GAG GC	HLA‐B, full length	3	NGS
B‐3’UTR‐3769	CTG CCC CAG CAC ACT GCA GC		In house	
C‐5’UTR fwd	TCA GGC ACA CAG TGT GAC AAA GAT	HLA‐C, full length	3	NGS
C‐3’UTR‐3779	CTG CAG CAC ACR ATC AGG TTT C		In house	
DQB1‐453 fwd	TGA CAG CAA TTT TCT CTC CCC TGA	HLA‐DQB1, full length	2	NGS
DQB1*04Ex1 fwd	ATG TCT TGG AAG AAG GCT TTG CG		In house	
DQB1‐6495 rev	TGG GGA TGA AAG GAG ATG ACC TG		2	
DRB1‐PE2‐F1	CTG CTG CTC CTT GAG GCA TCC ACA	HLA‐DRB1, 5’UTR‐Exon2	2	NGS
DRB1‐PE2‐F2	CTG CTA CTC CTT GAG GCA TCC ACA		2	
DRB1‐PE2‐F3	CTG CTG CTC CCT GAG GCA TCC ACA		2	
DRB1‐PE2‐R1	CTT CTG GCT GTT CCA GTA CTC GGC AT		2	
DRB1‐PE2‐R2	CTT CTG GCT GTT CCA GGA CTC GGC GA		2	
DRB1‐PE2‐R3	CTT CTG GCT GTT CCA GTA CTC AGC GT		2	
DRB1‐PE2‐R4	CTT CTG GCT GTT CCA GTA CTC CTC AT		2	
DRB1‐PE2‐R5	CTT CTG GCT GTT CCA GTG CTC CGC AG		2	
DRB1‐PE2‐R6	CTT CTG GCT GTT CCA GTA CTC GGC GC		2	
DRB1‐E2‐1.1‐F	GCA CGT TTC TTG TGG CAG CTT AAG TT	HLA‐DRB1, Exon2‐3’UTR	2	NGS
DRB1‐E2‐1.2‐F	GCA CGT TTC TTG TGG CAG CTA AAG TT		2	
DRB1‐E2‐2‐F	TTT CCT GTG GCA GCC TAA GAG G		2	
DRB1‐E2‐3568‐F	CAC AGC ACG TTT CTT GGA GTA CTC		2	
DRB1‐E2‐4‐F	AGC ACG TTT CTT GGA GCA GGT TAA ACA		2	
DRB1‐E2‐7‐F4	CAC AGC ACG TTT CCT GTG GCA GGG		2	
DRB1‐E2‐9‐F	CAC AGC ACG TTT CTT GAA GCA GGA		2	
DRB1‐E2‐10‐F	ACA GCA CGT TTC TTG GAG GAG GT		2	
DRB1‐E2‐12‐R	ATG CAC GGG AGG CCA TAC GGT		2	
DRB1‐E2‐3568‐R	ATG CAC AGG AGG CCA TAG GGT		2	
DRB1‐E2‐4‐R	ATG CAT GGG AGG CAG GAA GCA		2	
DRB1‐E2‐7‐R2	CAG ATG CAT GGG AGG CAG GAA GCG		2	
DRB1‐E2‐9‐R	ATG CAT GGG AGG CAG GAA GCG		2	
DRB1‐E2‐10‐R	TGG AAT GTC TAA AGC AAG CTA TTT AAC ATA TGT		2	
DRB1‐5’UTR	TCT GGC CCC TGG TCC TGT CCT GTT CTC CAG GG	HLA‐DRB1, full length	In house	NGS
DRB1‐3’UTR	TGC TGA ACC AGT AGC AAC CAG GTC C		In house	
DQA1fwd‐310	AGA CAT GCA CAC ACC AGA GAA GA	HLA‐DQA1, full length	In house	NGS
DQA1rev‐5441	TGC CAC TTC CCA ATT CCC CTA C		In house	
DPB1‐PRO‐F2	CCT CCT GAC CCT GAT GAC AGT CCT	HLA‐DPB1, 5’UTR‐Exon2	2	NGS
DPB1‐PRO‐R2	CCA TCT GCC CCT CAA GCA CCT CAA		2	
DPB1‐F2	CTC AGT GCT CGC CCC TCC CTA GTG AT	HLA‐DPB1, Exon2‐3’UTR	2	NGS
DPB1‐R2	GCA CAG TAG CTT TCG GGA ATT GAC CA		2	
DRB3‐In1fwd*01	GTG TGA CCG GAT CCT TCG TGT A	HLA‐DRB3, Intron1‐3’UTR	In house	NGS
DRB3‐In1fwd*02	GTG TGA CCG GAG CAT TCG TGT C		In house	
DRB3‐E2‐R1	ATG CAC AGG AGG CCA TAG GGT		2	
DRB5‐In1fwd	ATG GCG GCG TCT CTG TCA GTA	HLA‐DRB5, Intron1‐3’UTR	In house	NGS
DRB5‐E2‐R	ATG CAT GGG AGG CCG TAG GGT		2	
DRB4‐In1fwd	CCG GAT CGT TCG TGT CCC CA	HLA‐DRB4, Intron1‐3’UTR	In house	NGS
DRB4‐E2‐R	ATG CAT GGG AGG CAG GAC AGT		2	
DPA1‐F1	CTC TCT TGA CCA CGC TGG TAC CTA	HLA‐DPA1, full length	2	NGS
DPA1‐R1	TTG GCC TCT TGG CTA TAC CTC TTT T		2	
E‐fwd (E08072)	CAG CGT CGC CAC GAC TCC CGA C	HLA‐E, full length	4	NGS
E‐rev (E10034)	AGA CAC AGA GGT GGA CTG TTT CTC T		4	
G‐5’UTR260‐fwd	GAA GTC CCA GGG CCT CAA GC	HLA‐G, full length	In house	NGS
G‐rev 3228	CCC ATC AAT CTC TCT TGG AAA		In house	
MICA‐fwd Ex1	ACG CGT TGT CTG TCC TGG AA	MICA‐exon 1‐Exon2	5	NGS
MICA‐rev RG	CTA CGA CGG GGG TAA GGG AAG GGT T		6	
MICA‐fwd FG	CGT TCT TGT CCC TTT GCC CGT GTG C	MICA‐Exon2‐3’UTR	6	NGS
MICA‐rev 3’UTR	CGT GCC TGG CCT GAG ACT		7	
B. Primers used for sequencing				
B*44‐559 rev	TCG TCC ACG TAG CCC ACG GT	HLA‐B*44 559	In house	Sanger
B*44‐1034fwd	GGG TCT CAC ATC ATC CAG AGG	HLA‐B*44 1034	In house	Sanger
B*44‐1830fwd	GTC CTA GGG TGT CCC ATG AG	HLA‐B*44 1830	In house	Sanger
B*44‐2155rev	GAA GAG ATA TGA CCC CTC ATC C	HLA‐B*44 2155	In house	Sanger
B*44‐2182fwd	CTG GAG CCC TTC AGC AGG	HLA‐B*44 2182	In house	Sanger
B*44‐2346fwd	TGT GAT GTG TAG GAG GAA GAG C	HLA‐B*44 2346	In house	Sanger
B*44‐2797fwd	TCC CAG TCC CCT CAC AGG G	HLA‐B*44 2797	In house	Sanger
B*44‐3041rev	CCC ACC CAC CCC CAG ACC T	HLA‐B*44 3041	In house	Sanger
				

A. Lists all primers used for the generation of sequencing templates by PCR. B. Lists all sequencing primers that have been used for full length Sanger sequencing of the HLA‐B*44:138Q allele. In column “Designation” the names of the primers are listed; in column “Sequence (5′‐ >3′)” the sequence of the nucleotides is provided; in column “Coverage” the targeted gene and the location of the primers on this gene are indicated; in column “Reference” the origin of the primer sequence is listed: primers have been created by us (“in house”) or have been taken from publications; in column “Sequence technology” the technique used is stated. Oligonucleotides marked with *are also used as sequencing primers. The primers have been positioned to allow a full coverage of the allele

To further confirm these results, we performed full‐length sequencing of HLA‐B alleles from the patient and all available family members by NGS.^8^ This approach covered even longer regions of the 5’UTR and 3’UTR than we had obtained in the Sanger approach.

The family study confirmed the unequivocal segregation of the new allele ( Figure 1).

**Figure 1:**
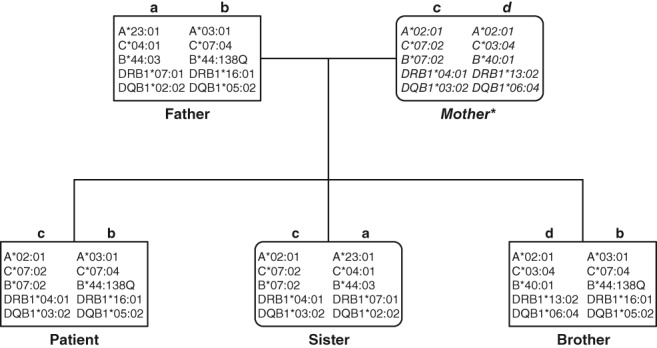
Pedigree chart of the patient's family; A‐D indicate HLA‐haplotypes; only HLA‐A, B, C, DRB1, and DQB1 genotypes at two field resolution are depicted for easy readability. The new allele is located on haplotype b*,* which is observed in the father and two siblings *the mother was at the time of typing already deceased, her haplotypes have been deduced. The typing has been performed by various methods:Serology: we used 180 in house validated typing sera to detect HLA class I molecules on the cell surface.Low resolution typing: SSO typing was performed for *HLA‐A*, ‐*B*, and ‐*C* genes using a commercial reverse dot blot assay (Dynal, Bloomsborough, UK), SSP typing was performed for the *HLA B* gene only using a commercial SSP assay (Genovision, Vienna, UK).Sequencing‐based typing is described in Figure 2.The full characterisation of HLA‐haplotypes is as follows*:* Haplotype a: G*01:04:04, A*23:01:01, E*01:01:01:01, C*04:01:01:01, B*44:03:01:01, MICA*004, DRB4*01:01:01:01, DRB1*07:01:01:01, DQA1*02:01:01:01, DQB1*02:02:01:01, DPA1*01:03:01:04, DPB1*04:01:01:01. Haplotype b: G*01:01:01:05, A*03:01:01:01, E*01:03:02:01, C*07:04:01:01, B*44:138Q, MICA*008:01:02, DRB5*02:02, DRB1*16:01:01, DQA1*01:02:02, DQB1*05:02:01, DPA1*01:03:01:02, DPB1*04:01:01:01. Haplotype c: G*01:01:01:01, A*02:01:01:01, E*01:01:01:01, C*07:02:02:03, B*07:02:01, MICA*008:04, DRB4*01:03:01:01, DRB1*04:01:01:01, DQA1*03:01:01, DQB1*03:02:01, DPA1*01:03:01:02, DPB1*04:01:01:01. Haplotype d: G*01:01:01:01, A*02:01:01:01, E*01:03:02:01, C*03:04:01:01, B*40:01:02, MICA*008:04, DRB3*03:01:01, DRB1*13:02:01, DQA1*01:02:01:04, DQB1*06:04:01, DPA1*01:03:01:01, DPB1*02:01:02:06

To further characterise the haplotypes, we typed other class I genes, HLA‐A, C, E, and G and class II genes, HLA‐DRB1, DRB3/4/5, DQA1, DQB1, DPA1, and DPB1; additionally the class I‐related *MICA* genes were typed. All of those genes were typed at full length. The results are provided in Figure 1. All alleles observed have been listed in the database; it thus seems that the events, leading to the generation of the new HLA‐B allele did not affect other functional genes of the haplotype.

Restricting the view on the sequence to exons 1 to 3, the relationship of the novel allele with the HLA‐B*44 group seemed apparent: sequences were identical except the deletion of three nucleotides. After we had obtained the full length sequence, it became evident, that the region from the 5′ end to Intron 3 matched perfectly with HLA‐B*44:02:01:01 and other HLA‐B*44 alleles. From position 1620 in exon 4, however, several mismatches appeared; this part of the sequence matched perfectly with HLA‐B*15:18:01:02 (Figure 2), although. From position 1221 in intron 3 to position 1619 in exon 4 both putative founder alleles share the sequence, a recombination between the two alleles might therefore have occurred there.^9^ By contrast, the deletion of three nucleotides in exon 3 is a unique feature of HLA‐B*44:138Q, indicating an independent event, that led to the creation of this allele.

**Figure 2:**
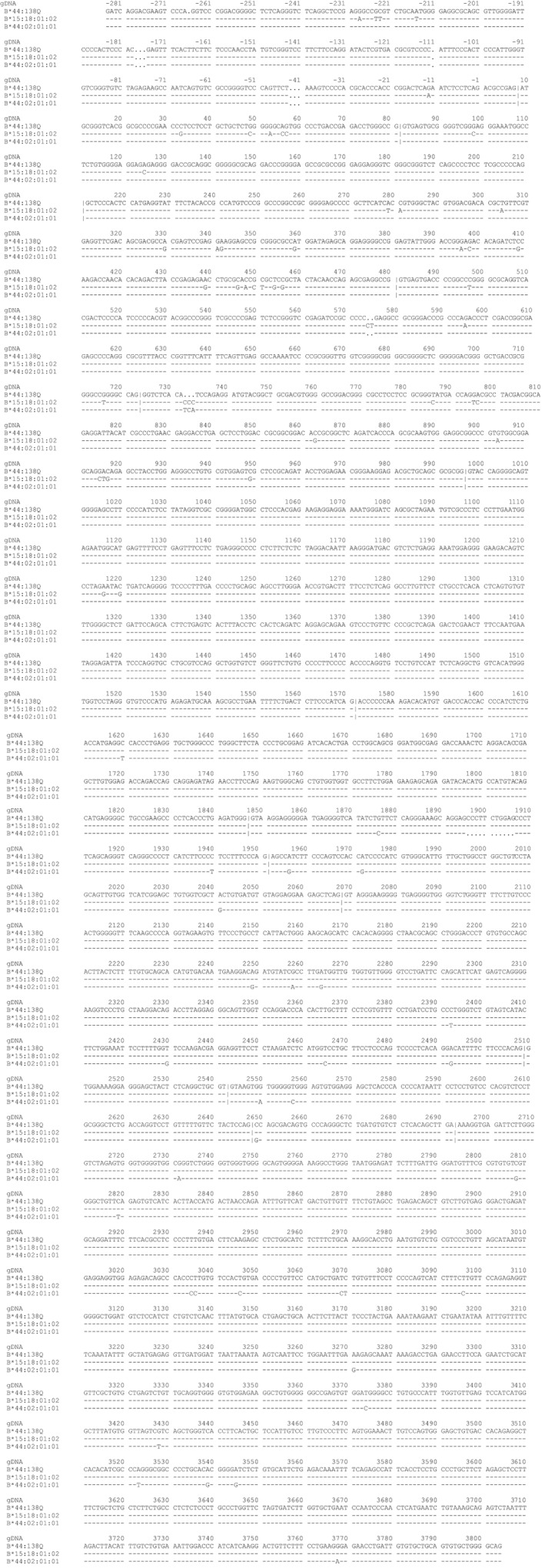
Sequence alignments of HLA‐B*44:138Q, HLA‐B*44:02:01:01, and HLA‐B*15:18:01:02. Alignments have been generated using the tool of the IMGT/HLA database (https://www.ebi.ac.uk/cgi‐bin/ipd/imgt/hla), version 3.33.0. Bases identical with HLA‐B*44:138Q are indicated by dashes, different nucleotides are marked, and deletions are signposted with dots; “|” denotes exon/intron borders. The numbering of base positions in the genomic DNA (gDNA) is according to the IMGT/*database.* The HLA‐B*44:138Q sequence was obtained by nucleotide Sanger sequencing (positions 22 to 456) and whole gene Sanger sequencing (positions −10 to 3034) or NGS (positions −284 to 3804). For the preparation of an amplification template for Sanger sequencing, primers for whole gene amplification were designed that covered the gene from its 5’UTR to the 3’UTR and separated HLA‐B*07 and HLA‐B*44 allele groups. For amplification the PCR Qiagen Long‐Range PCR Kit (Qiagen GmbH Hilden, Germany) was used. Sequencing was performed with overlapping primers for the whole HLA‐B*44 allele. All primers are listed in Table 1. Cycle sequencing was performed using a Big Dye Terminator Cycle Sequencing kit (ABI, Foster City, CA). The sequencing products were analysed on an ABI 3100 capillary sequencer. For confirmation, heterozygous PCR products spanning exons 1 to exons 3 were cloned with TA Cloning kit pCR 2.1 vector (Invitrogen, Carlsbad, USA) and also subjected to Sanger sequencing. For NGS analyses, long‐range amplification of the whole *HLA‐B* gene was achieved with the primers listed in Table 1. Amplification was performed using GoTaq Long PCR Mastermix (Promega Corporation, Woods Hollow Roads Madison). Library preparation was performed according to the manufacturers' instructions. Size selection was performed on an E‐Gel (Invitrogen, Kiryat Shoma, Israel); only fragments with sizes >400 bp were selected. After quantification, 26 PMol of fragments were used for emulsion PCR. Enriched Ion sphere particles were loaded onto a Ion Torrent 316 chip v2 (Life Technologies, Carlsbad, California) and subsequently sequenced on an Ion PGM device (LifeTechnologies, Carlsbad, California) with a flow number of 800, for 400 base reads. Analysis of the reads was performed using two different NGS analysis software packets (TypeStream NGS Analysis Software, One Lambda, Inc. Canoga Park CA; NGSengine, GenDX, Utrecht, The Netherlands)

In this respect, the new allele differs from the majority of other HLA alleles, where a simple “fixation of a single recombination event was responsible for the origin”^9^; whether these events have occurred simultaneously or in independent meioses remains impossible to decide.

Because of the fact that this deletion comprised three nucleotides and occurred within two identical neighbouring codons (ATC‐ATC) the resulting protein was shortened by just one isoleucin. This deletion concerns the second domain at the edge between the α‐helix and the β‐sheet. It is not likely that this position is part of an epitope.^10^, ^11^ However, the conformation of the molecules might change which could involve modifications of serological epitopes. This assumption would be in concordance with results from lymphocytotoxic tests: sera specific for HLA‐B44 (n = 2), HLA‐B12 (n = 2), or Bw4 (n = 3) did not show any reactivities with cells of the HLA‐B*44:138Q‐positive individual.

From a technical point, the analysis of the full‐length gene by NGS was much simpler than the Sanger approach, because of the clonal nature of the NGS sequencing, there was no necessity to separate alleles beforehand and no additional sequencing primers had to be designed. The sequence of the HLA‐B*44:138Q allele was concordant with the Sanger result except a homopolymer at position 3061 of the 3’UTR: With the Sanger technique, 3 cytosins were detected while the consensus of the NGS analysis indicated only 2 cytosins. Given that Sanger sequencing has represented the golden standard for HLA typing for decades^12^ this discrepancy argues for a good concordance of the new technology, albeit we have compared only one of the several platforms.

In summary, the characterisation of HLA‐B*44:138Q on the IonTorrent platform allowed a fast analysis of whole genes with much less effort compared with Sanger sequencing. However, the homopolymer issue^13^ of NGS remains a diagnostic challenge. The generation of the new allele within a haplotype that otherwise consists of common HLA‐alleles (as assessed by full length sequencing) indicates that the mechanisms leading to this new allele are restricted to a single gene.

## CONFLICTS OF INTEREST

The authors have declared no conflicting interests.
